# PPAR agonists as therapeutics for CNS trauma and neurological diseases

**DOI:** 10.1042/AN20130030

**Published:** 2013-12-18

**Authors:** Shweta Mandrekar-Colucci, Andrew Sauerbeck, Phillip G. Popovich, Dana M. McTigue

**Affiliations:** *Center for Brain and Spinal Cord Repair, Department of Neuroscience, Wexner Medical Center at The Ohio State University, Columbus, OH 43210, U.S.A.

**Keywords:** Alzheimer’s disease, astrocyte, experimental autoimmune encephalomyelitis (EAE), macrophage, multiple sclerosis, spinal cord injury, ALS, amyotrophic lateral sclerosis, Arg1, Arginase 1, BMP, bone morphogenetic protein, 15d-PGJ2, 15-deoxy-Δ-12,14-prostaglandin J-2, EAE, experimental autoimmune encephalomyelitis, GR, glucocorticoid receptor, IL, interleukin, iNOS, inducible nitric oxide synthase, LPS, lipopolysaccharide, MS, multiple sclerosis, NF-κB, nuclear factor κB, NGF, nerve growth factor, OPC, oligodendrocyte precursor cell, PPAR, peroxisome proliferator-activated receptor, RXR, retinoid X receptor, SCI, spinal cord injury, SHP-2, Src homology region 2-containing protein tyrosine phosphatase-2, TBI, traumatic brain injury, Th1, T helper type 1, TNFα, tumour necrosis factor α, UCP, uncoupling protein

## Abstract

Traumatic injury or disease of the spinal cord and brain elicits multiple cellular and biochemical reactions that together cause or are associated with neuropathology. Specifically, injury or disease elicits acute infiltration and activation of immune cells, death of neurons and glia, mitochondrial dysfunction, and the secretion of substrates that inhibit axon regeneration. In some diseases, inflammation is chronic or non-resolving. Ligands that target PPARs (peroxisome proliferator-activated receptors), a group of ligand-activated transcription factors, are promising therapeutics for neurologic disease and CNS injury because their activation affects many, if not all, of these interrelated pathologic mechanisms. PPAR activation can simultaneously weaken or reprogram the immune response, stimulate metabolic and mitochondrial function, promote axon growth and induce progenitor cells to differentiate into myelinating oligodendrocytes. PPAR activation has beneficial effects in many pre-clinical models of neurodegenerative diseases and CNS injury; however, the mechanisms through which PPARs exert these effects have yet to be fully elucidated. In this review we discuss current literature supporting the role of PPAR activation as a therapeutic target for treating traumatic injury and degenerative diseases of the CNS.

## INTRODUCTION

Neurodegenerative diseases [e.g. MS (multiple sclerosis), ALS (amyotrophic lateral sclerosis), and Alzheimer's disease] and traumatic or ischemic CNS injuries [e.g. SCI (spinal cord injury), stroke, TBI (traumatic brain injury)] all elicit neuroinflammatory cascades. Specifically, the collective effects of activated glia, inflammatory cytokines and chemokines, a compromised blood–brain/spinal cord barrier, and infiltrating leukocytes exacerbate axon damage and demyelination, mitochondrial dysfunction, and glial scar formation. The result is a tissue environment that favors cell death and inhibits mechanisms of endogenous repair (Norenberg et al., 2004; Fleming et al., 2006; Popovich and Longbrake, 2008). Since mature CNS neurons are post-mitotic and regenerate poorly, the destructive effects of trauma, disease and neuroinflammation render affected individuals permanently disabled.

PPARs (peroxisome proliferator-activated receptors) comprise a family of ligand-activated transcription factors that play a vital role in cellular processes such as cell differentiation and metabolism (Kersten et al., 2000; Bensinger and Tontonoz, 2008). They also are potent regulators of macrophage differentiation that, when activated, can attenuate pathology associated with various chronic neuroinflammatory diseases (Odegaard et al., 2008; Bouhlel et al., 2009; Chawla, 2010;). PPARs exist as three different isoforms, α, δ (also called β), and γ, and all are expressed by microglia, astrocytes, neurons and oligodendrocytes, albeit at different levels (Kliewer et al., 1994).

PPARs form obligate heterodimers with RXRs (retinoid X receptors), and ligand binding to either PPAR or RXR initiates gene transcription. PPAR–RXR heterodimers are termed ‘permissive’ because ligation of either component of the heterodimer can induce transcriptional activation of the receptor complex. This means that PPAR activation can be induced to varying degrees by ligands activating RXRs. Since the precise mechanisms by which RXR ligands affect PPAR signaling are not yet defined, it is important to note that RXR activation may not be identical with direct PPAR activation. Currently, FDA-approved agonists of PPARα and PPARγ are used to treat hyperlipidemia and Type II diabetes, respectively (Table 1). These same drugs are also ideal candidates for translational research in models of CNS trauma and disease (Lehmann et al., 1995; Staels et al., 1998).

**Table 1:** Commonly used PPAR agonists for CNS studies This table is a list of commonly utilized PPAR agonists that have been tested experimentally to attenuate neurological disease/injury. Instances of clinical use in humans are restricted to the following neurological conditions: Alzheimer's disease, multiple sclerosis, stroke, amyotrophic lateral sclerosis and Parkinson's disease.

Drug (other names)	Receptor target	FDA approved	Prescribed treatment	Toxicity/side effects	Clinical trial for CNS disease/trauma	References
Pioglitazone (Actos)	PPARγ	Yes	Type 2 diabetes	Associated with bladder tumors. Weight gain	Multiple sclerosis, Alzheimer's disease, stroke amyotrophic lateral sclerosis Parkinson's disease	Wilcox et al., 2007; Hanyu et al., 2009; Kaiser et al., 2009; Shukla et al., 2010; Geldmacher et al., 2011; Sato et al., 2011; Dupuis et al., 2012
Rosiglitazone (Avandia)	PPARγ	Yes	Type 2 diabetes	Increased cardiovascular risk	Alzhiemer's disease	Watson et al., 2005; Kume et al., 2012
Troglitazone (Rezulin, Resulin, Romozin, Noscal)	PPARγ	Formerly, taken off the market by FDA	Type 2 diabetes	Liver toxicity	N/A	
15-deoxy-Delta(12,14)-prostaglandin J(2)	PPARγ	No			N/A	
Telmisartan (Micardis)	PPARγ/PPARδ	Yes	Hypertension	Tacy/bradycardia, edema, hypotension	Alzheimer's disease, stroke	Diener et al., 2008; Yusuf et al., 2008; Bath et al., 2009; Kume et al., 2012
Gemfibrozil (Lopid, Jezil, Gen-Fibro)	PPARα	Yes	Hyperlipidemia	Gastrointestinal distress, musculoskeletal pain, gallstones, increased risk of cancer, reduced blood K^+^ levels	N/A	
Fenofibrate (Tricor, Trilipix)	PPARα	Yes	Hyperlipidemia	Gastrointestinal distress, skin reactions, severly reduced high-density lipoprotein levels	N/A	

*In vivo* studies document that agonists for different PPAR isoforms typically improve outcomes in pre-clinical models of CNS injury or disease. For instance, in EAE (experimental autoimmune encephalomyelitis, an animal model of MS), several PPAR agonists have proven effective in delaying the onset and progression of disease (Niino et al., 2001; Diab et al., 2002, 2004; Feinstein et al., 2002; Gocke et al., 2009). PPARδ and PPARγ agonists also have shown benefits in experimental models of SCI, TBI and stroke (McTigue et al., 2007; Yi et al., 2008; Allahtavakoli et al., 2009; Sauerbeck et al., 2011; Thal et al., 2011; Villapol et al., 2012). Activation of these receptors attenuated inflammation and apoptosis, reduced lesion size and improved functional recovery; they also promoted oligodendrogenesis and differentiation (McTigue et al., 2007; Park et al., 2007; Yi et al., 2008; Allahtavakoli et al., 2009; Paterniti et al., 2010; Meng et al., 2011; Sauerbeck et al., 2011; Thal et al., 2011; Villapol et al., 2012). Neuropathology was exacerbated after CNS injury in mice deficient in PPARα, suggesting that endogenous PPAR ligands may limit neuropathology (Genovese et al., 2005). PPARα activation facilitated recovery after TBI, but surprisingly had no effect or worsened recovery after SCI (Besson et al., 2005; Chen et al., 2007, 2008; Almad et al., 2011) suggesting that PPAR activation may not be uniformly beneficial.

In animal models of ALS, a disease that causes paralysis and eventual death due to loss of upper and lower motor neurons, PPARγ agonists extend survival and attenuate motor neuron loss (Kiaei et al., 2005; Shibata et al., 2008). However, in a phase II double-blind controlled clinical trial, the PPARγ agonist pioglitazone did not increase survival in ALS patients (Dupuis et al., 2012).

Activation of PPARs also yielded conflicting data in rodent models of Alzheimer's disease and in human subjects. For example, in some, but not all studies, PPAR activation reduced amyloid deposition and reversed cognitive and memory decline (Yan et al., 2003; Pedersen and Flynn, 2004; Heneka et al., 2005; Nicolakakis et al., 2008; Escribano et al., 2010; Toledo and Inestrosa, 2010; Mandrekar-Colucci et al., 2012). The inconsistencies in the reported data may be due to use of different animal models of Alzheimer's disease, poor blood–brain barrier penetrance of PPAR agonists (i.e. inconsistent drug distribution) and widely variable dosing strategies (Maeshiba et al., 1997; Hemauer et al., 2010). Phase III clinical trials testing another PPARγ agonist, rosiglitazone, failed to show efficacy in patients with mild to moderate stages of Alzheimer's disease; however, the doses used in clinical trials were significantly lower than those shown to be beneficial in the rodent models (Gold et al., 2010).

**Figure 1:**
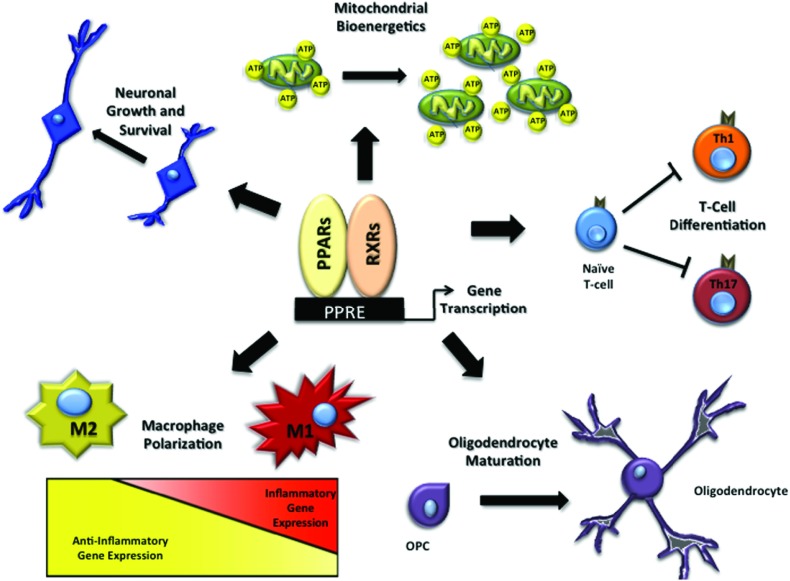
PPARs modulate multiple pathways in the CNS PPAR activation after CNS injury/trauma promotes recovery through multiple mechanisms by promoting (1) axon outgrowth, (2) mitochondrial bioenergetics, (3) inhibition of Th1 and Th17 T-cell differentiation, (4) OPC maturation, and (5) polarization of macrophages from an inflammatory ‘M1’ to an anti-inflammatory ‘M2’ activation state.

Clearly, PPAR activation has the potential to be beneficial in many neuropathological conditions. The mechanisms of action of PPAR agonists are so diverse that they may be advantageous at many stages of injury. Thus, the best timing and dose of agonists may vary depending on injury severity, progression of disease or the cellular target (i.e. neurons, microglia, oligodendrocytes), and may explain the conflicting results in studies listed above. A clearer understanding of how and where PPARs act will facilitate designing the most effective pre-clinical and clinical studies. This review will address the many mechanisms through which PPAR activation is known to alleviate pathology and improve neurological function in the damaged CNS (Figure 1).

## PPARs AND MACROPHAGE POLARIZATION

Microglia are the primary immune effector cells of the CNS. Pathological changes in the brain or spinal cord cause rapid microglial migration to the affected area where they undergo phenotypic and morphologic transformation. If sufficiently activated, these cells also release chemotactic and inflammatory cytokines that signal the recruitment of monocytes from the circulation into the pathological CNS (Davalos et al., 2005; Nimmerjahn et al., 2005).

The phenotype and corresponding function of macro-phages and microglia are shaped by a cadre of signals present in pathological tissue (Gordon and Martinez, 2010). These signals collaborate to instruct a population of cells that, at any given time, can be quite heterogeneous. To simplify this intrinsic complexity, working models of microglia and macrophage function are often used in which the cells are broadly defined using nomenclature and phenotypic signatures developed from *in vitro* models. For example, ‘classically’ activated M1 macrophages and ‘alternatively’ activated M2 macrophages are distinct macrophage subsets that can be generated *in vitro* using defined stimuli (Mosser and Edwards, 2008). Classical activation of macrophages is associated with antigen presentation and the production of inflammatory cytokines, chemokines and reactive oxygen species. Chronic persistence of M1 macrophages is thought to exacerbate disease and tissue pathology (Horn et al., 2008; Busch et al., 2009; Kigerl et al., 2009; Martinez et al., 2009; Hu et al., 2012). In contrast, M2 macrophages produce immune regulatory cytokines including TGF-β (transforming growth factor β), IL (interleukin)-10, IL-13 and IL-4 as well as wound healing molecules such as Arg1 (Arginase 1), YM1, MR (mannose receptor, CD206) and FIZZ1 (RELMα).

Using canonical molecular indicators of macrophage phenotype, recent studies have identified M1 and M2 macrophages in the pathological brain and spinal cord (Kigerl et al., 2009; Mandrekar-Colucci and Landreth, 2010; Kumar et al., 2013). In many models of neurologic disease, the magnitude of pathology or functional loss correlates with a robust M1 macrophage response, and blocking inflammatory signaling often confers neuroprotection (Colton and Wilcock, 2010; David and Kroner, 2011; Shechter and Schwartz, 2013). Similarly, functional inhibition or acute depletion of macrophages in rats and mice after traumatic SCI is neuroprotective and promotes functional recovery (Giulian and Robertson, 1990; Blight, 1994; Popovich et al., 1999; Gris et al., 2004)

Recent data indicate that PPARγ and PPARδ are critical transcriptional ‘gatekeepers’, i.e. they control transcriptional modules that influence macrophage phenotype. PPAR activation inhibits the expression of M1 genes in cells exposed to M1-type stimuli and enhances the expression of M2 markers in the presence of M2 stimuli. In particular, activation of these PPARs in macrophages induces the M2 markers Arg1, CD206, YM1 and FIZZ1; these effects are lost in mice deficient for either receptor (Bouhlel et al., 2007; Odegaard et al., 2007, 2008; Gallardo-Soler et al., 2008; Kang et al., 2008). PPAR activation produces similar effects in microglia (Storer et al., 2005a; Xu et al., 2005b; Ramanan et al., 2009; Antonietta Ajmone-Cat et al., 2012). For instance, PPAR activation of microglia promotes phagocytosis of pathological protein aggregates and is neuroprotective in models of MS and Alzheimer's disease (Mandrekar-Colucci et al., 2012; Yamanaka et al., 2012). Activation of all three PPAR isoforms inhibits NF-κB (nuclear factor κB)-mediated induction of inflammatory cytokine genes (Chawla, 2010). PPARγ achieves this through ligand-activated sumolyation of the receptor, which then binds to and stabilizes the interaction between NF-κB and its co-repressor complex, thereby preventing the transcription of inflammatory cytokines (Pascual et al., 2005).

In Alzheimer's disease, which is characterized by chronic neuroinflammation, PPARγ activation attenuates neuroinflammation and augments expression of M2 macrophage markers, indicating that peripheral administration of PPAR agonists can influence an active and chronic inflammatory milieu in the CNS (Mandrekar-Colucci et al., 2012). PPAR activation is beneficial in other pathological conditions including TBI, SCI, EAE, stroke and ALS (Kiaei et al., 2005; Schutz et al., 2005; Drew et al., 2006; Sundararajan et al., 2006; Yi et al., 2008; Villapol et al., 2012). In EAE, infiltration of monocytes correlates with progression to the severe paralytic stages of disease (Ajami et al., 2011). Treating EAE animals with PPAR agonists is anti-inflammatory and slows disease progression; however, whether PPARs act solely by altering macrophage polarization in this model has not been confirmed (Niino et al., 2001; Diab et al., 2002, 2004; Feinstein et al., 2002; Gocke et al., 2009).

The molecular phenotype of microglia or macrophages affects the ability of these cells to phagocytose debris. For instance, activation of PPARδ in macrophages promotes clearance of apoptotic cells (Mukundan et al., 2009). This occurs through increased expression of opsonins (e.g. complement C1q) by macrophages, which increases phagocytosis of apoptotic cells (Mukundan et al., 2009). Similarly, PPARγ activation in microglia promotes phagocytosis by up-regulating the scavenger receptor CD36 (Yamanaka et al., 2012). Considering traumatic CNS injuries produce large amounts of myelin and cell debris, PPAR-induced M2 polarization of macrophages and microglia may be beneficial by promoting removal of debris in addition to the other mechanisms mentioned above.

Finally, it should be noted that while the *in vitro*-derived M1 and M2 nomenclature is widely used to describe macrophage activation states, these classifications are imperfect and reflect only a subset of states existing on a continuum of macrophage activation (Mosser and Edwards, 2008). Thus, characterizing M1 as ‘bad’ and M2 as ‘good’ macrophages is overly simplistic since both types have important functions and it is likely that an imbalance in their ratios causes pathology, especially if the imbalance is prolonged. Chronic inflammation involving both M1 and M2 macrophages is documented in many CNS diseases and injuries such as Alzheimer's disease, MS, SCI, TBI, and stroke (Colton et al., 2006; Kigerl et al., 2009; Mikita et al., 2011; Hu et al., 2012; Kumar et al., 2013). Therapeutically targeting PPARs may help to ‘re-balance’ these two phenotypes in the injured CNS and promote neuroprotection.

## PPARs AND ASTROCYTES

Astrocytes are highly reactive cells, and in the pathological state, they can release damaging molecules that cause neuron loss (Bal-Price and Brown, 2001). The ability of astrocytes to promote inflammation and their responsiveness to PPAR agonists positions these cells to play a critical role in the progression and treatment of neurological disease. PPAR agonists attenuate pathological astrocyte activation and improve disease progression (Diab et al., 2002, 2004; Storer et al., 2005a, 2005b; Mandrekar-Colucci and Landreth, 2010; Hong et al., 2012). Given that astrocytes play an important role in most CNS disorders, targeting them with PPAR agonists may prove effective in multiple settings.

Astrocytes can regulate how they respond to PPAR ligands through changes in receptor expression. In LPS (lipopolysaccharide)-stimulated astrocytes, PPARγ activation leads to a positive feed-forward signal that increases expression of PPARδ, and PPARδ activation increases expression of PPARα (Aleshin et al., 2009). In turn, PPARα provides a negative-feedback signal inhibiting PPARδ expression (Aleshin et al., 2009). This coordinated signaling helps regulate how PPAR activation influences inflammation (Aleshin et al., 2009). Since the environment surrounding astrocytes can change dramatically with injury or disease, the ability for astrocytes to alter their responses to PPAR ligands allows more precise control of inflammation.

Modulating inflammation is one of the best-studied roles of PPAR activation in astrocytes and has been examined in multiple experimental models (Diab et al., 2002; Giri et al., 2004; Storer et al., 2005a, 2005b; Xu and Drew, 2007; Xu et al., 2007; Lee et al., 2008; Tjalkens et al., 2008; Pineau et al., 2010; Cowley et al., 2012; Hong et al., 2012). In the spinal cord, the PPARγ agonist pioglitazone reduces astrocyte activation in a receptor-dependent manner (Jia et al., 2013). Similarly, the PPARγ agonists 15d-PGJ2 (15-deoxy-Δ-12,14-prostaglandin J-2) and rosiglitazone, and PPARα agonists gemfibrozil and fenofibrate reduced levels of the IL-12 family of cytokines, nitric oxide, IL-6, IL-1β and MCP-1 in primary astrocyte cultures exposed to LPS (Xu and Drew, 2007; Xu et al., 2006, 2007). It is important to note that each drug has different effects on cytokine expression, which can be advantageous since similarly acting drugs can be combined for more potent effects (Diab et al., 2004). Furthermore, attenuating inflammatory signals reduces disease severity in models of MS, even after the onset of clinical symptoms (Diab et al., 2002, 2004). Notably, these beneficial effects can occur through PPAR-dependent and PPAR-independent mechanisms. For example, pioglitazone reduced intraspinal astrocyte activation in a receptor dependent manner in the sciatic nerve transection model (Jia et al., 2013), while 15d-PGJ2, another PPARγ agonist, promoted astrocyte-mediated neuroprotection independent of PPARγ (Giri et al., 2004; Haskew-Layton et al., 2013).

In models of Alzheimer's disease, activation of PPARs in astrocytes is protective against amyloid-β accumulation (Kalinin et al., 2009; Valles et al., 2010; Wang et al., 2010; Benito et al., 2012; Mandrekar-Colucci et al., 2012). Given the well-established effects of PPAR ligands in other cell types, it is not surprising that these effects occur both through astrocytes and microglia (Wang et al., 2010; Mandrekar-Colucci et al., 2012). The ability of astrocytes to attenuate amyloid-β-induced toxicity depends on the activation and presence of PPARs (Valles et al., 2010; Benito et al., 2012). Exposing astrocytes with reduced PPAR expression to amyloid-β exacerbated production of the inflammatory molecules TNFα (tumour necrosis factor α), IL-6, iNOS (inducible nitric oxide synthase), and COX-2 (cyclo-oxygenase 2) compared with wild-type astrocytes (Benito et al., 2012). Furthermore, treating with a PPARα or PPARγ agonist attenuated the increased inflammatory response in amyloid-β-treated astrocytes (Benito et al., 2012). Encouragingly, the reduced amyloid-dependent toxicity led to improved cognition (Mandrekar-Colucci et al., 2012).

## PPARs AND T-CELL ACTIVATION

T-cells cross the blood–brain/spinal barrier and secrete various cytokines, including IFNγ (interferon γ), IL-17, and TNFα, all of which can damage myelin and neurons. These processes play an integral role during neurological insult. For example, MS is mediated primarily by autoreactive T-cells of the Th1 (T helper type 1) or Th17 phenotype (Trinchieri et al., 2003; Fletcher et al., 2010). Given that T-cells express PPARα and PPARγ, these PPARs can influence the adaptive immune system by modifying the activity of these cells (Marx et al., 2002). Several studies show that agonists for all three PPAR isoforms inhibit Th1-cell expansion and cytokine production, and in some cases, can concomitantly increase expression of Th2 cytokines (Niino et al., 2001; Diab et al., 2002, 2004; Feinstein et al., 2002; Gocke et al., 2009; Kanakasabai et al., 2010). This may explain why Th1 responses are enhanced in PPARγ-deficient mice, and EAE pathology is exacerbated in mice treated with PPARγ antagonists (Natarajan et al., 2003; Raikwar et al., 2005).

## PPARs AND OLIGODENDROCYTES

Oligodendrocytes are the myelinating cells of the CNS and are highly susceptible to various components of the pathologic cascades that occur in most or all neurological diseases (McTigue and Tripathi, 2008). Oligodendrocytes are vulnerable to inflammatory mediators (e.g. cytokines and chemokines) and because of their high intracellular iron and low levels of antioxidant molecules, they are exquisitely sensitive to oxidative damage from reactive oxygen and nitrogen species (Thorburne and Juurlink, 1996; Juurlink et al., 1998). Loss of myelinating oligodendrocytes exposes axons to the injury milieu, which can lead to axon degeneration and, in some cases, neuronal death.

Within the CNS, NG2+ OPCs (oligodendrocyte precursor cells) can differentiate into mature myelinating oligodendrocytes following injury or demyelination (McTigue and Tripathi, 2008). However, the mechanisms that regulate these processes after injury or insult are not well understood (McTigue et al., 1998; McTigue and Tripathi, 2008; Whittaker et al., 2012). In models of MS, clinical symptoms and demyelination are exacerbated in PPARγ heterozygous mice (Natarajan et al., 2003). Work by De Nuccio et al. (2011) points to a potential mechanism; they show that PPARγ agonists promote OPC differentiation by inducing mitochondrial respiratory chain activity and oscillatory Ca^2+^ waves, which are crucial for oligodendrocyte differentiation. Furthermore, PPARγ activation directly promotes differentiation of rat OPCs into mature oligodendrocytes (Bernardo et al., 2009). Since myelin is composed mostly of lipid and since PPARs play a major role in lipid metabolism, it is not surprising that PPARs regulate the differentiation and function of oligodendrocytes (Saluja et al., 2001; Leisewitz et al., 2008; Kanakasabai et al., 2012). Statins (cholesterol-reducing drugs) also promote oligodendrocyte maturation by inducing PPARγ, an effect that is blocked by PPARγ antagonism (Sim et al., 2008). Thus, targeting oligodendrocytes through PPARs will likely enhance the production and maturation of OPCs, repopulate lost oligodendrocytes and maintain myelination and the integrity of axons during CNS pathology.

**Figure 2:**
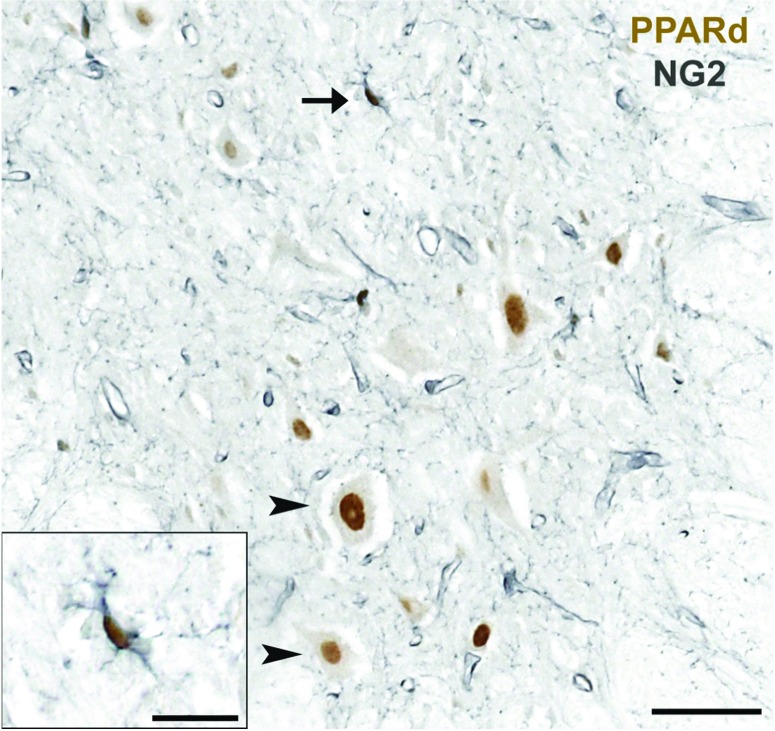
NG2+ oligodendrocyte progenitor cells express PPARδ Spinal cord section from a normal rat spinal cord gray matter (ventral horn) immunolabeled for PPARδ (brown) and NG2 (gray), a marker for oligodendrocyte progenitor cells. In the normal spinal cord, PPARδ is expressed by NG2 cells (arrow, inset) and is also visible in NG2-negative motor neurons (arrowheads). Scale bar=50 μm; scale bar in inset=20 μm.

Like PPARγ, PPARδ also appears to be important in oligodendrocyte lineage cell regulation. It is expressed by OPCs in the adult CNS (Figure 2) and, after SCI, the number of PPARδ-expressing OPCs increases along the lesion border where robust oligodendrocyte genesis occurs (Tripathi and McTigue, 2007; Almad and McTigue, 2010). In EAE, PPARδ promotes oligodendrocyte differentiation by limiting the effects of BMPs (bone morphogenetic proteins). Oligodendrocytes express BMPs and their receptors, and during CNS development, BMPs restrict OPC maturation (Gross et al., 1996; Hardy and Friedrich, 1996). PPARδ activation counteracts BMP signaling by increasing the expression of noggin, a BMP antagonist produced by astrocytes. In the presence of noggin, BMPs are inhibited and the number of myelin-producing oligodendrocytes is increased (Simonini et al., 2010). Thus, these findings further indicate a direct role for PPARδ in the regulation of oligodendrocytes.

Even with evidence showing a role for PPARs in oligodendrocyte regulation, conflicting data regarding the efficacy of these agonists on promoting OPC maturation or differentiation have been documented. In one study, undifferentiated C6 glioma cells were shown to up-regulate oligodendrocyte markers in response to agonists that target PPARγ, but not PPARδ or PPARα. In the same study, however, overexpression of PPARδ committed C6 cells to oligodendrocyte fate through the up-regulation of PPARγ (Leisewitz et al., 2008). These studies suggest that oligodendrocyte differentiation may depend on the coordinate activation of PPARs.

Activating PPARs may also enhance oligodendrocyte survival in the injured or diseased CNS. Oligodendrocytes express all three PPAR isoforms and PPAR activation promotes differentiation and myelin gene expression (Granneman et al., 1998; Saluja et al., 2001; Roth et al., 2003; Woods et al., 2003; Jana et al., 2012). Indeed, genes for many of the myelin proteins contain PPAR response elements indicating they can be directly targeted by PPARs (Jana et al., 2012). For example, the PPARα agonist gemfibrozil stimulates expression of the myelin genes MBP (myelin basic protein), MOG (myelin oligodendrocyte glycoprotein), PLP (proteolipid protein) and CNPase. Using chromatin immunoprecipitation assays, it is possible to show that gemfibrozil enhances binding of PPARδ rather than PPARα, its receptor target, to promoters of myelin genes in human oligodendrocytes (Jana et al., 2012). Moreover, activation of PPARδ promotes myelin protein expression (Saluja et al., 2001).

Additionally, CNS pathology can alter PPAR expression by oligodendrocyte lineage cells. For instance, PPARδ expression is enhanced in OPCs and oligodendrocytes after SCI, suggesting these cells would be responsive to PPAR signaling (Almad and McTigue, 2010). Indeed, activation of PPARγ and PPARδ after SCI decreases lesion area, increases myelination and promotes locomotor activity (McTigue et al., 2007; Park et al., 2007; Paterniti et al., 2010). PPARγ activation also protects myelin in an *in vitro* model of inflammatory demyelination (Paterniti et al., 2010). Similarly, PPAR activation in the EAE model delays onset and reduces the severity of clinical symptoms (Niino et al., 2001; Diab et al., 2002; Feinstein et al., 2002; Genovese et al., 2005; Gocke et al., 2009).

Enhanced myelination and oligodendrocyte survival following PPAR activation could occur independent of changes in myelin gene expression. PPAR activation suppresses synthesis of inflammatory cytokines/chemokines and reactive oxygen (ROS) and nitrogen (RNS) species, all of which are toxic to oligodendrocytes (Springer et al., 1997; Zhao et al., 2006; McTigue, 2008). These inflammatory mediators are inhibited by PPAR activation in macrophages and astrocytes (Ricote et al., 1998; Bernardo et al., 2000; Xu et al., 2006). PPARγ activation increases cellular antioxidants, including catalase and copper-zinc superoxide dismutase, both of which are expressed at low levels in oligodendrocyte lineage cells (Juurlink et al., 1998; Bernardo et al., 2009). Treating OPCs with the PPARγ antagonist GW9662 abolished the anti-inflammatory and antioxidant effects of PPAR agonists, demonstrating a direct role for PPAR signaling in these signaling cascades (Bernardo et al., 2009).

Oligodendrocytes are vulnerable to glutamate excitotoxicity (McAdoo et al., 1999; Pitt et al., 2000; Xu et al., 2005a). High levels of extracellular glutamate are believed to contribute to several neurological diseases and after CNS injury (McAdoo et al., 1999; Pitt et al., 2000; Bogaert et al., 2010; Hinzman et al., 2010, 2012; Thomas et al., 2012; Mehta et al., 2013). Indeed, glutamate antagonists are neuroprotective in many pre-clinical models of neurologic disease (Wrathall et al., 1997; Rosenberg et al., 1999; Faden et al., 2001). Like traditional glutamate antagonists, PPAR activation may also attenuate excitotoxicity. For instance, the PPARγ agonist rosiglitazone increases expression of the glutamate transporter GLT1/EEAT2 mRNA and protein in cultured astrocytes (Romera et al., 2007). An increase in functional GLT1 would promote glutamate uptake by astrocytes thereby reducing extracellular levels. However, when tested *in vivo* in a model of focal cerebral ischemia, rosiglitazone did not affect GLT1/EAAT2 expression (Verma et al., 2011). Thus, the effects may be context-dependent or require a more rigorous analysis of dosing schedule or pharmacokinetics.

Collectively, data from these studies suggest that PPARs likely act in concert to promote oligodendrocyte survival and OPC differentiation and may represent a novel molecular target, that, if activated appropriately, could promote oligodendrocyte replacement and remyelination in the injured or diseased CNS.

## PPARs: NEURON SURVIVAL AND AXON REGENERATION

Neuron loss is a devastating and permanent effect of CNS trauma or disease. Several studies have shown neuroprotective effects of PPARs, most notably PPARγ. For instance, lipid peroxidation was shown to raise PPARγ levels in motor neurons in a model of ALS (Benedusi et al., 2012). This was believed to be a self-protective mechanism since PPARγ activation promotes the expression of lipid detoxifying genes such as lipoprotein lipase and glutathione transferase a-2 (Benedusi et al., 2012). PPARγ also may regulate neuronal responses to ischemia since conditional deletion of PPARγ in neurons increases their susceptibility to ischemia *in vitro* (Zhao et al., 2009). Further, the PPARγ agonist troglitazone enhances survival of rat motor neurons in culture and PPARγ activation by 15d-PGJ2 protects PC12 cells from nitrosative-induced cell death (Lim et al., 2004). *In vivo*, activating PPARγ in a middle cerebral artery occlusion model of stroke reduces infarct size and lowers cyclin D1, a protein involved in programmed cell death (Pei et al., 2010). Additionally, PPARγ activation can stabilize mitochondria and protect neurons against apoptotic cell death and oxidative stress by upregulating the anti-apoptotic protein bcl-2 (Fuenzalida et al., 2007). At least part of the neuroprotective effects of PPARγ involves synergistic signaling with neurotrophins. For instance, NGF (nerve growth factor)-induced neuronal differentiation is mediated through activation of PPARγ in a TrkA-dependent manner. Further, PPARγ activation increases NGF and BDNF levels after SCI (Fuenzalida et al., 2005; Meng et al., 2011). Together these studies suggest that activation of PPARs, and in particular PPARγ, may be neuroprotective and promote neuronal survival.

Injured axons have a limited capacity to spontaneously regenerate. Therefore, interventions that enhance or stimulate axon growth may further increase recovery or minimize the functional deficits caused by CNS injury. A few studies have reported that PPAR activation promotes axonal growth in neuronal cell lines and primary DRG (dorsal root ganglion) neuron cultures. Specifically, the PPARγ agonists pioglitazone and 15d-PGJ2 increase the number and lengths of neurites (Jung et al., 2003; Miglio et al., 2009). These effects may occur through modulating RhoA, which is increased in injured neurons and limits axon regeneration after CNS injury (Dubreuil et al., 2003; Madura et al., 2004). Ibuprofen (which activates PPARγ at micromolar levels) inhibits RhoA and stimulates corticospinal and serotonergic axon sprouting after spinal cord transection in rats (Lehmann et al., 1997; Fu et al., 2007). Work by others showed that the growth-promoting effects of ibuprofen involved PPARγ activation and its ability to inhibit RhoA activation (Dill et al., 2010). This effect may be mediated by SHP-2 (Src homology region 2-containing protein tyrosine phosphatase-2), which is involved in the PPARγ-dependent inhibition of RhoA (Wakino et al., 2004). However, the potential role of SHP-2 in PPAR-mediated neurite outgrowth has not yet been studied in the CNS. Like their ability to promote neuronal survival, these studies show that PPAR activation can positively affect axon regeneration.

## PPARs AND NEUROPATHIC PAIN

Neuropathic pain is a debilitating consequence of CNS injury, MS, and other neurological diseases. Currently, there is no cure for chronic neuropathic pain and most analgesics are ineffective. Interestingly, emerging pre-clinical data indicate that agonists for PPARα and PPARγ attenuate neuropathic pain following peripheral nerve injury (Churi et al., 2008; Maeda et al., 2008; Taylor, 2009; Takahashi et al., 2011; Ruiz-Medina et al., 2012). The anti-inflammatory effects of PPAR activation, occurring through genomic and non-genomic PPAR-dependent mechanisms, may mediate such effects. For example, intrathecal injection of the PPARγ agonists 15d-PGJ2 or rosiglitazone rapidly (<5 min) attenuated pain-like behaviors in rodents; the effects were PPARγ-dependent since co-administering PPARγ antagonists blocked the effects (Churi et al., 2008). It is thought that because actions occur within minutes of drug administration that these analgesic effects must be non-genomic (Fehrenbacher et al., 2009).

PPARs also may affect pain sensations by modulating glucocorticoid action. Glucocorticoids are steroid hormones released during periods of acute or sustained stress, both physiological and psychological, and signal via the GR (glucocorticoid receptor), another nuclear receptor. Glucocorticoids can pass the blood–brain/spinal barrier and excess levels are detrimental to neuronal survival in the brain, inducing synaptic loss, atrophy of the hippocampus and cognitive deficits. In the CNS, glucocorticoid signaling enhances pain-like behaviors and is up-regulated in parallel with inflammatory cytokines after injury (Blackburn-Munro and Blackburn-Munro, 2003). PPARγ activation inhibits the autonomic and neuroendocrine responses to stress in rats and may explain why activation of this receptor reduces circulating corticosterone levels (Ryan et al., 2012). A 5-day treatment with rosiglitazone attenuates corticosterone levels, heart rate, and expression of c-Fos (a marker of neuronal activation) in the hypothalamus of rats that were subjected to restraint stress (Ryan et al., 2012). Rosiglitazone also decreased circulating corticosterone levels in a mouse model of Alzheimer's disease (Escribano et al., 2009). While the mechanisms through which PPARs attenuate corticosterone remain to be fully elucidated, PPARα activation does interfere with GR-dependent gene expression by blocking the recruitment of RNA polymerase II to the glucocorticoid response elements on the promoter of GR target genes (Bougarne et al., 2009). While the effects of glucocorticoids after injury are complicated, it is apparent in these studies that PPARs play an integral role in this signaling pathway.

## PPARs AND MITOCHONDRIAL BIOENERGETICS

Mitochondrial dysfunction is common in the CNS with stroke, SCI, TBI, ALS, MS, Huntington's disease and Alzheimer's disease (Mecocci et al., 1996; Mattiazzi et al., 2002; Wiedemann et al., 2002; Korde et al., 2005, 2007; Vijayvergiya et al., 2005; Dutta et al., 2006; Singh et al., 2006; Sullivan et al., 2007; Robertson et al., 2007; Regenold et al., 2008; Vyshkina et al., 2008; Martin et al., 2009; Pandya et al., 2009; Patel et al., 2009; Readnower et al., 2011; Sauerbeck et al., 2011; Zhao et al., 2011; Lunnon et al., 2012). In these conditions, mitochondrial dysfunction correlates with cell death, functional impairment, and cognitive deficit. This is intuitively obvious since energy production by mitochondria is essential for survival of all cells, including neurons (Nicholls and Budd, 2000; Stephans et al., 2002; Borland et al., 2008). Oligodendrocytes also have high energy demands since they must maintain large amounts of plasma membrane as myelin. Damage to oligodendrocyte mitochondria impairs energy metabolism resulting in reduced myelin production and compaction, and ultimately hypo-myelination or complete axon demyelination (Kalman et al., 1997). Accordingly, finding new therapies that protect mitochondria should help protect neurons and oligodendrocytes in most, if not all, forms of CNS disease.

PPAR agonists have been extensively studied for their role in modulating metabolism and energy production (Alaynick, 2008; Sugden et al., 2009). Activation of PPAR receptors by fatty acids promotes mitochondrial β-oxidation allowing for greater cellular energy production (Gulick et al., 1994). Many of the effects of PPARs on bioenergetics occur through regulation of gene expression. Specifically, activation of PPARδ increases production of mTFA (mitochondrial transcription factor), UCPs (uncoupling proteins) 2 and 3 (UCP2/3), and lipoprotein lipase (Muoio et al., 2002; Dressel et al., 2003; Jiang et al., 2010). Similarly, PPARα activation increases the transport and utilization of fatty acids needed during β-oxidation and PPARγ activation increases cytochrome *c* oxidase 6A2 (Desvergne and Wahli, 1999; Allen et al., 2006). Activation of PPARγ also induces the expression of lipoprotein lipase and stimulates mitochondrial biogenesis (Strum et al., 2007; Benedusi et al., 2012; Morino et al., 2012). Further, PPARγ activation stabilizes existing mitochondria and prevents their dysfunction (Fuenzalida et al., 2007; Quintanilla et al., 2008). These effects may underlie the increased mitochondrial energy production observed following administration of PPARγ agonists in models of CNS insult (Hunter et al., 2007; Sauerbeck et al., 2011).

In addition to driving gene transcription, some PPAR agonists interact directly with mitochondria (Colca et al., 2004; Geldenhuys et al., 2010). These effects can occur via mitoNEET, a protein in the mitochondrial outer membrane that is essential for maximal energy production (Wiley et al., 2007). Pioglitazone binds to mitoNEET and stabilizes its conformational structure (Colca et al., 2004; Paddock et al., 2007). The mitochondrial effects of pioglitazone likely extend to other PPAR agonists such as rosiglitazone, which also binds to mitoNEET (Geldenhuys et al., 2010; Bieganski and Yarmush, 2011). Given this novel direct mitochondrial target for PPAR agonists, work has focused on creating specific ligands for mitoNEET (Geldenhuys et al., 2010, 2011; Bieganski and Yarmush, 2011). These new ligands may prove effective at targeting mitochondrial dysfunction and improving recovery similar to traditional PPAR ligands.

## RXRs AS A MEANS TO TARGET PPARs

RXRs are essential for PPAR signaling. Specifically, RXRs heterodimerize with PPARs, creating ‘permissive’ signaling complexes that increase expression of PPAR target genes following ligation with either a RXR-specific agonist or a PPAR partner ligand (Mangelsdorf and Evans, 1995; Szanto et al., 2004). There are three RXR isotypes: RXRα, RXRβ and RXRγ. In the intact CNS, neurons and glia constitutively express RXRs (Schrage et al., 2006). In injury or disease, the subcellular location of RXR switches from the cytoplasm to the nucleus, suggesting transcriptional activation of RXR-containing heterodimers (Schrage et al., 2006). Known ligands for RXRs include honokiol (a naturally occurring ligand from the bark of the magnolia tree), the synthetic agonist Bexarotene (Targretin), and 9-cis retinoic acid (Qu and Tang, 2010; Kotani et al., 2010). Considering that Bexarotene is already FDA-approved and has an excellent side-effect profile, it is an optimal candidate for translational studies on neurodegenerative diseases or injuries (Lansigan and Foss, 2010).

Activation of RXR elicits a response similar to that observed after PPAR activation. For instance, RXR activation promotes an anti-inflammatory milieu by down-regulating inflammatory signaling in microglia and astrocytes (Xu and Drew, 2006). It also can initiate oligodendrocyte progenitor proliferation, differentiation and myelination (Chao et al., 2010; Nunez et al., 2010; Huang et al., 2011; Kaushik et al., 2012). Interestingly, transcripts for all RXRs are highly up-regulated in demyelinated lesions, with the RXRγ isoform being the highest of the three (Huang et al., 2011). Furthermore, 9-cis retinoic acid enhances OPC differentiation in culture and increases remyelination in cerebellar slice cultures (Huang et al., 2011). Thus, RXR activation could be therapeutic in demyelinating diseases. Additionally, their enhanced ability to readily cross the blood–brain barrier, compared with popular PPAR agonists, makes RXR agonists attractive candidates for the treatment of neurologic diseases (Cramer et al., 2012).

Moreover, due to the promiscuous nature of RXR heterodimer activation, PPAR signaling pathways may be initiated through the use of RXR agonists. Thus, RXR agonists could benefit any disease in which PPAR activation has proven effective. This promiscuity also creates unique challenges and opportunities. Since these signaling cascades may be differentially activated based on the binding specificity and affinity of various ligands to the receptor, RXR activation may not mimic the spectrum of changes that occur when PPAR-specific agonists are used to activate the heterodimer complex. Currently, it is not known which heterodimeric partner RXR exerts its beneficial effects through. Thus, a level of precision concerning RXR signaling is missing. Given the similarity of actions between PPAR and RXR activation, RXR activation may be exerting its effects by concurrently activating multiple PPAR pathways. Also, studies have shown that PPAR and RXR agonists, when used together to simultaneously activate the heterodimer complex, have synergistic effects allowing for maximal stimulation and expanding possible treatment paradigms (Papi et al., 2009; Yamanaka et al., 2012).

## NON-TRADITIONAL ACTIONS OF PPARs

PPAR agonists can influence pathological processes through mechanisms that are independent of their classical PPAR receptors. For example, when given at extremely low doses (0.5 and 1 mg/kg), far below those needed to activate PPARγ receptors, pioglitazone still attenuates inflammatory signaling by reducing TNF-α, iNOS, and IL-1β (Thal et al., 2011). Indeed, co-administration of a PPARγ antagonist does not prevent the anti-inflammatory effects of low-dose pioglitazone, confirming a PPARγ receptor-independent mechanism (Thal et al., 2011). Similarly, although pioglitazone reduces tissue loss and cognitive impairment after TBI by PPARγ activation, this drug reduces microglial activation via a PPARγ-independent mechanism (Sauerbeck et al., 2011). A different PPARγ agonist, rosiglitazone, has similar anti-inflammatory effects after TBI, yet its effects depend on PPARγ activation (Yi et al., 2008). The ability to reduce inflammation and the different receptor dependency of pioglitazone and rosiglitazone is likely explained by immune cells expressing both PPARγ and PPARδ receptors and each receptor having different thresholds for activation by rosiglitazone (Sakamoto et al., 2000; Gordon and Martinez, 2010). Additionally, the PPARγ-independent actions of these agonists likely result from their ability to directly target mitochondria and also activate other PPAR receptors (Sakamoto et al., 2000; Colca et al., 2004; Paddock et al., 2007; Orasanu et al., 2008; Geldenhuys et al., 2011). Evidence of receptor-dependent and independent effects, especially within the same animal, provides strong support for the diverse nature of the beneficial effects of PPAR agonists.

## CONCLUSIONS

The beneficial effects of PPAR activation have been independently reproduced in many rodent models of traumatic injury and neurodegenerative disease and there are several potential mechanisms through which PPAR activation promotes CNS repair and functional recovery. Activation of PPARs can reduce inflammation and confer neuroprotection, in part through their ability to minimize cell death and reduce mitochondrial dysfunction. PPAR activation may also enhance axonal growth and remyelination. Through non-genomic mechanisms, PPAR agonists may have analgesic effects. Since the pathophysiology of traumatic CNS injury and neurodegeneration is dynamic, the timing of PPAR activation likely needs to be tailored to meet the specific characteristics of the disease in question. Still, the broad effects on overlapping mechanisms of neurologic injury make these drugs very promising therapeutics for treating traumatic injuries to the brain or spinal cord as well as various neurodegenerative diseases.
